# Predicting COVID-19 Incidence Using Wastewater Surveillance Data, Denmark, October 2021–June 2022

**DOI:** 10.3201/eid2908.221634

**Published:** 2023-08

**Authors:** Oliver McManus, Lasse Engbo Christiansen, Maarten Nauta, Lene Wulff Krogsgaard, Naja Stolberg Bahrenscheer, Lene von Kappelgaard, Tobias Christiansen, Mikkel Hansen, Nicco Claudio Hansen, Jonas Kähler, Anders Rasmussen, Stine Raith Richter, Lasse Dam Rasmussen, Kristina Træholt Franck, Steen Ethelberg

**Affiliations:** European Centre for Disease Prevention and Control, Solna, Sweden (O. McManus);; Statens Serum Institut, Copenhagen, Denmark (O. McManus, L.E. Christiansen, M. Nauta, L.W. Krogsgaard, N.S. Bahrenscheer, L. von Kappelgaard, T. Christiansen, M. Hansen, N.C. Hansen, J. Kähler, A. Rasmussen, S.R. Richter, L.D. Rasmussen, K.T. Franck, S. Ethelberg);; University of Copenhagen, Copenhagen, Denmark (S. Ethelberg)

**Keywords:** COVID-19, wastewater surveillance, SARS-CoV-2, coronavirus disease, severe acute respiratory syndrome coronavirus 2, viruses, respiratory infections, zoonoses, epidemiological monitoring, epidemiological models, epidemiological methods, epidemiology, time series, Denmark

## Abstract

Analysis of wastewater is used in many settings for surveillance of SARS-CoV-2, but it remains unclear how well wastewater testing results reflect incidence. Denmark has had an extensive wastewater analysis system that conducts 3 weekly tests in ≈200 sites and has 85% population coverage; the country also offers free SARS-CoV-2 PCR tests to all residents. Using time series analysis for modeling, we found that wastewater data, combined with information on circulating variants and the number of human tests performed, closely fitted the incidence curve of persons testing positive. The results were consistent at a regional level and among a subpopulation of frequently tested healthcare personnel. We used wastewater analysis data to estimate incidence after testing was reduced to a minimum after March 2022. These results imply that data from a large-scale wastewater surveillance system can serve as a good proxy for COVID-19 incidence and for epidemic control.

The COVID-19 pandemic has shown the need for accurate surveillance data. Incidence rate data, commonly collected as part of human surveillance, can only be interpreted with the understanding that local testing strategies vary over time. COVID-19 surveillance using wastewater testing, in which SARS-CoV-2 RNA fragments shed in the feces of infected persons are quantified, has been implemented in many countries (*1*–*5*). Wastewater data have been suggested as a complement to or even a substitute for human surveillance data, particularly in times of low human testing activity. The association between wastewater concentrations and incidence has been demonstrated in multiple settings, but few studies have succeeded in predicting incidence through wastewater surveillance, and the direct value of wastewater testing for epidemic control remains debatable (*1*).

In response to the pandemic, Denmark set up an extensive wastewater surveillance system, which was implemented in July 2021 and fully rolled out in October 2021. During the study period, the system included 201 wastewater treatment plant (WWTP) inlets, which were sampled 3 times a week and covered 85% of the population. Denmark has also had exceptionally high COVID-19 testing capacity, offering unlimited, free reverse transcription PCR (RT-PCR) testing through public testing stations (*6**,**7*; M.A. Gram et al., unpub. data, http://medrxiv.org/lookup/doi/10.1101/2023.02.06.23285556). The per capita testing rate has been among the highest in the world during some periods of the pandemic; the country tested up to 27% of the population per week in December 2021 and was capturing an estimated 70% of active COVID-19 cases at the start of 2022 (M.A. Gram et al., unpub. data). However, testing activity was scaled down in early 2022, to <1% per week by June 2022 (*8*,*9*).

Given the variation in testing rates, wastewater concentrations should not be directly compared with observed incidence. Instead, models should include information on changing testing rates over time. Another strategy is to look at a subgroup of regularly tested persons, where the effect of fluctuations in testing patterns should be less pronounced. Such a subgroup exists in Denmark, where recommendations were made for regular screening tests for certain care personnel (*10*).

The association between wastewater data and incidence might be affected by the SARS-CoV-2 variants in circulation, because those variants could have different fecal shedding patterns. Viral load for oropharyngeal samples has been shown to be higher for Delta than previous variants (B. Li et al., unpub. data, https://www.medrxiv.org/content/10.1101/2021.07.07.21260122v2), but how fecal shedding differs among variants is not known (*11*). Other variables, such as temperature and traveling time of SARS-CoV-2 in sewers, dilution by precipitation or wastewater from industry, and inhibitors of laboratory analyses, might affect viral quantification, (*12*,*13*).

We used the results of wastewater surveillance to predict the observed incidence of SARS-CoV-2 infections in Denmark. We performed the analysis at the national and regional level and among a subgroup of healthcare personnel.

## Methods

### Overview

We conducted a time-series analysis, constructing a model to explain observed incidence by wastewater concentrations. Besides the main national-level analysis, we also tested the model at a regional level and on a subpopulation of healthcare personnel. We used the human testing rate as a covariate in our model and considered interactions between wastewater concentrations and the proportion of circulating Omicron versus Delta variants and between wastewater concentrations and wastewater temperature. The study period was September 27, 2021–June 26, 2022.

### Data Sources

#### Wastewater

Throughout the study period, 24-hour composite samples were taken 3 times a week from 202 WWTP inlets across Denmark. Sampling started on Mondays, Tuesdays, and Thursdays. Where possible, the samples were flow-proportional, which enabled sampling of more water at times of heavy flow, providing a more representative sample of the 24-hour water flow. Otherwise, samples were time-proportional, sampling a fixed amount of water at fixed time intervals.

Samples were purified and analyzed using quantitative real-time RT-PCR at Eurofins Miljø, a central commercial laboratory in Vejen, Denmark. We measured cycle threshold (Ct) values for 2 SARS-CoV-2 genes (the N2 region of the nucleocapsid gene and the RNA-dependent RNA polymerase [RdRp]) and converted them to concentrations (copies/L). We calculated limits of detection (LOD) and limits of quantification (LOQ) for each gene in each sample. We imputed values <LOD as LOD/2, and <LOQ as (LOD + LOQ)/2. Starting in 2022, we also measured the concentrations of 2 indicators of fecal concentration: crAssphage and pepper mild mottle virus (*14*,*15*). For consistency with 2021 data, we used those measurements as data quality indicators but not to normalize SARS-CoV-2 concentrations.

For each sample, utility companies reported the volume of wastewater that entered WWTPs over the 24-hour sampling window and the temperature of wastewater upon entry. Utility companies also provided geographic information, which we used to calculate the resident population of each catchment area by linking to the Danish Civil Registration System (*16*).

#### Incidence

In-person PCR COVID-19 testing was available for free to all residents throughout our study period; results were collected centrally in the Danish Microbiology Database (*17*). Testing recommendations changed throughout the study period; the most substantial change occurred on March 10, 2022. After that date, tests for the general population were recommended only for symptomatic persons in groups at high risk (*9*). We extracted data on daily incidence of PCR-confirmed COVID-19 cases and weekly PCR testing rate from Denmark’s official COVID-19 statistics (*18*) for its 5 administrative regions.

#### Care Personnel

We used data on healthcare personnel, consisting of care home staff and in-home caretakers, for a secondary analysis. During September 4, 2021–April 28, 2022, weekly PCR tests were recommended for healthcare personnel for screening purposes. After this time and until the end of the study period, the recommendation was 1 test every 2 weeks (*10*). Because of those recommendations, we believed incidence in this group might be less affected by testing patterns and therefore a better measure of actual community incidence than observed incidence among the general population. Information on this group came from Denmark’s centrally collected data on COVID-19 tests and results, linked to employment information through the Civil Registration System (*16*,*19*).

#### Variants

Denmark had extensive whole-genome sequencing to monitor SARS-CoV-2 variants, sequencing up to 15,000 samples/week (*20*,*21*). We calculated the weekly proportion of sequences belonging to the Delta and Omicron variants through the start of June 2022, excluding other variants that were present in negligible amounts.

### Data Processing

**Figure 4:**
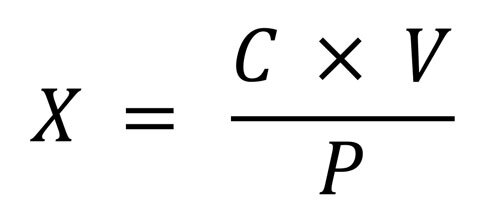
Equation for the average number of SARS-CoV-2 RNA copies shed per person living in the WWTP catchment area during a 24-hour sampling period.

Our wastewater measure for each sample is expressed as the average number of SARS-CoV-2 RNA copies shed per person living in the WWTP catchment area during a 24-hour sampling period. We calculated this value using the equation (Figure 4)

where C is the geometric mean of the N2 and RdRp gene concentrations (in copies/L) and is log-normally distributed, V is the volume of wastewater (in liters) that entered the WWTP in a 24-hour measurement period, and P is the population size of the catchment area. We removed outliers of wastewater volume measurements, defined as being >1.5 times the interquartile range from the 25th or 75th percentile, and then truncated values at 1.96 SDs from the mean on the log scale.

We aggregated all data into weekly observations: incidence was weekly cases per 100,000 population; testing rate was weekly tests per 1,000 population; wastewater concentrations were the weekly weighted median of all average copies per person measurements (≈600 values of *X* per week), using the log_10_-transformed population size of each WWTP catchment area as the weights (the choice of weight being a compromise between equal weighting because of the uncertainty of individual measurements and weighting according to population size); wastewater temperature was similarly the weekly weighted median. Wastewater concentrations were log-normally distributed, and the variance of incidence and testing rates increases with the values, so we used log_10_-transformed versions of those variables in our models, using the same transformation for all to ease interpretation.

#### Exclusions

Because great fluctuations in the fecal load of wastewater are not expected, finding such a fluctuation in measurements of the fecal indicators pepper mild mottle virus and crAssphage indicated a likely failure in sampling or laboratory analysis. We therefore excluded samples with missing or extreme concentrations of these fecal indicators, defined as concentrations >3 SDs from the mean for each WWTP, on the log scale. We also excluded samples that the laboratory received on unexpected days of the week. Such samples might not have been comparable to others because more time had passed in which RNA content could have degraded during transit. Furthermore, we excluded samples with missing values for the volume of wastewater entering the WWTP over a 24-hour sampling period. Finally, we excluded samples from WWTPs where we had no geographic information defining the catchment area, which we needed to define the population served by each WWTP. We also discarded wastewater temperature data reported as <1°C or >30°C, but we did not exclude other data for those samples from the analysis.

### Statistical Analysis

First, we plotted the national incidence and wastewater concentrations to compare patterns visually. Second, we fitted a model to see whether wastewater results were a predictor of national incidence. We split the data into a training and testing set. We used training data from before June 9, 2022, to select and estimate our models. We reserved data from June 9, 2022, onward (7 weekly datapoints) as an out-of-sample test dataset for model validation. We constructed an ARIMAX (autoregressive integrated moving average with exogenous variables) model, using incidence as the dependent variable and wastewater concentrations and testing rate as the explanatory variables. We tested including the interaction between wastewater concentrations and the circulating variant (expressed as proportion of Delta sequences) and the interaction between wastewater concentration and wastewater temperature. We only included temperature as an interaction with the wastewater concentration to restrict it to describe degradation of RNA and not the overall seasonal effect on incidence. Likewise, the proportion of Delta was included as an interaction with the wastewater concentration to adjust for different shedding patterns for the different variants. We selected which covariates to include based on the Akaike information criterion. For consistency, we used the terms selected for the national model in the secondary models as well. Third, we also estimated the model allowing for a time delay between wastewater results and incidence. We examined lag times of 0, 1, and 2 weeks in each direction, comparing the resulting models by the Akaike information criterion. Fourth, we validated the model on the out-of-sample dataset not used in the model estimation, using the root mean squared error. Fifth, we repeated the modeling steps in 2 secondary analyses: reestimating the main model independently for each region and for the subpopulation of care personnel only. For the care personnel model, we used both incidence and testing rate specific to care personnel. Sixth, we used the national model to predict the incidence that would have been observed had the testing rate remained stable throughout the study period by generating model predictions where we fixed the testing rate at a constant value. We used the highest recorded testing rate in the study period for this. We used R version 4.1.3 (The R Project for Statistical Computing, https://cran.r-project.org) for statistical analyses (Appendix).

## Results

### Description of Data

We included 18,737 wastewater samples from 202 WWTPs in the study (Appendix Table 1). Our initial dataset consisted of 21,069 samples, but we excluded 2,361 samples (29 for extreme concentrations of fecal indicators, 301 from WWTPs with unknown populations, 81 that arrived on unexpected days of the week, 919 missing data on wastewater flow in the sampling period, and 1,031 with sampling method not listed as flow-proportional or time-proportional). Of the included samples, 15,801 were flow-proportional and 2,764 were time-proportional. After aggregating by week at national level, we had 39 wastewater data points. We included a median of 515 (interquartile range 480.5–532.5) weekly samples.

**Figure 1:**
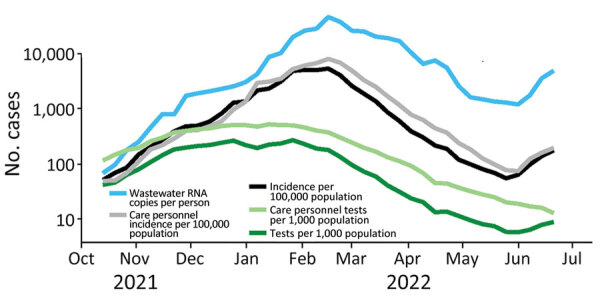
Comparison of results of COVID-19 wastewater surveillance with incidence and testing rate (both national and for care personnel) over time, Denmark, illustrating the agreement between wastewater concentrations and incidence. Wastewater concentrations are based on 18,565 individual samples. Testing rates were high during November 2021–February 2022 and decreased after that time.

Wastewater concentrations and incidence followed similar patterns, increasing until early 2022, decreasing until late May, and then increasing again (Figure 1). Testing rates remained fairly stable during November 2021–February 2022, after which they decreased to low levels. Per person, on average 2.6 times as many weekly tests occurred among healthcare personnel as occurred among the general population.

Incidence followed a similar pattern in all regions (Appendix Figure 1), although slightly offset in time. Numbers throughout 2021 were slightly higher in the Capital region and neighboring Zealand region than in the other 3 regions. The pattern of testing rate over time also did not differ greatly by region (Appendix Figure 2).

Until late November 2021, nearly all human isolates sequenced were the Delta variant. Omicron quickly took over in December, reaching ≈50% halfway through the month and >95% in the first week of January 2022. After that, nearly all samples were Omicron (Appendix Figure 3).

### Model Results

Our final models were based on wastewater concentration, testing rate, and the interaction of wastewater concentration with circulating variants (percentage Delta). They did not include the interaction between wastewater results and wastewater temperature, because it did not improve the model (Appendix). The model performed best with no lead or lag time between wastewater results and incidence.

**Figure 2:**
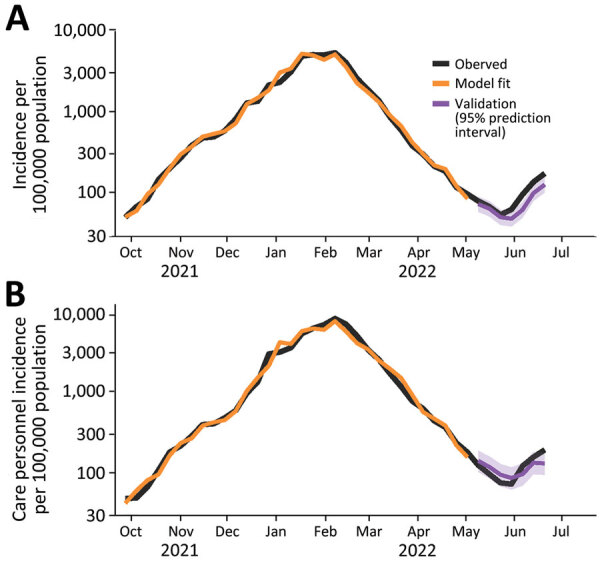
Model fit and forecasts for the national model (A) and the care personnel model (B) used in study of predicting COVID-19 incidence using wastewater surveillance data, Denmark. The fitted values (orange) are seen to follow the observed incidence (black) during the training period. The forecasts in the test period (purple) are also shown against the observed incidence (black).

The pattern of the model fit and validation estimates follows the pattern of observed incidence well (Figure 2; Appendix Figure 4). However, only 43% of validation points were covered by the 95% prediction intervals in the national model (Appendix Table 3).

The coefficients for wastewater results were generally 0.4–0.5 during Omicron; coefficients were lower during Delta by ≈0.15–0.20 (Table). The coefficient for wastewater was higher in the care personnel model (0.52 [95% CI 0.46–0.59] during Omicron) than in the main national model (0.40 [95% CI 0.34–0.46]). At a regional level, the wastewater coefficient was lower for the Capital region than other regions (0.31 [95% CI 0.24–0.38] during Omicron).

**Table:** Estimated coefficients from the national model, care personnel model, and regional model in study using wastewater surveillance data to predict COVID-19 incidence, Denmark, October 2021–June 2022*

Model	Term	Estimate (95% CI)		p value
National	AR (*1*)	0.46 (0.16–0.76)		0.004
	Wastewater concentration	0.4 (0.34–0.46)		<0.001
	Testing rate	0.87 (0.81–0.94)		<0.001
	Wastewater concentration × Delta (%)	−0.15 (−0.19 to −0.11)		<0.001
	Intercept	−0.14 (−0.32 to 0.04)		0.12
Care personnel	AR (*1*)	0.32 (−0.06 to 0.70)		0.1
	Wastewater concentration	0.52 (0.46–0.59)		<0.001
	Testing rate (care personnel)	0.84 (0.73–0.94)		<0.001
	Wastewater concentration × Delta (%)	−0.17 (−0.21 to −0.13)		<0.001
	Intercept	−2.45 (−2.83 to −2.07)		<0.001
Regional				
Capital Region	AR (*1*)	0.31 (−0.07 to 0.70)		0.12
	Wastewater concentration	0.31 (0.24–0.38)		<0.001
	Testing rate	1.03 (0.94–1.11)		<0.001
	Wastewater concentration × Delta (%)	−0.15 (−0.19 to −0.12)		<0.001
	Intercept	−0.11 (−0.34 to 0.12)		0.3
Central Denmark	AR (*1*)	−0.19 (−0.56 to 0.18)		0.3
	Wastewater concentration	0.48 (0.45–0.52)		<0.001
	Testing rate	0.88 (0.84–0.92)		<0.001
	Wastewater concentration × Delta (%)	−0.14 (−0.16 to −0.12)		<0.001
	Intercept	−0.48 (−0.60 to −0.36)		<0.001
North Denmark	AR (*1*)	−0.09 (−0.46 to 0.28)		0.6
	Wastewater concentration	0.47 (0.43–0.51)		<0.001
	Testing rate	0.91 (0.86–0.96)		<0.001
	Wastewater concentration × Delta (%)	−0.16 (−0.19 to −0.13)		<0.001
	Intercept	−0.49 (−0.64 to −0.33)		<0.001
Southern Denmark	AR (*1*)	0.16 (−0.19 to 0.51)		0.4
	Wastewater concentration	0.48 (0.44–0.52)		<0.001
	Testing rate	0.83 (0.78–0.88)		<0.001
	Wastewater concentration × Delta (%)	−0.15 (−0.17 to −0.12)		<0.001
	Intercept	−0.39 (−0.52 to −0.26)		<0.001
Zealand	AR (*1*)	0.07 (−0.34 to 0.47)		0.7
	Wastewater concentration	0.42 (0.37–0.48)		<0.001
	Testing rate	0.8 (0.73–0.86)		<0.001
	Wastewater concentration × Delta (%)	−0.14 (−0.17 to −0.11)		<0.001
	Intercept	−0.11 (−0.26 to 0.04)		0.2

*The response variable incidence, wastewater concentration, and testing rate are included after log_10_-transformation. AR (1) denotes the first-order autoregressive term.

### Predicted Incidence at Stable Testing

**Figure 3:**
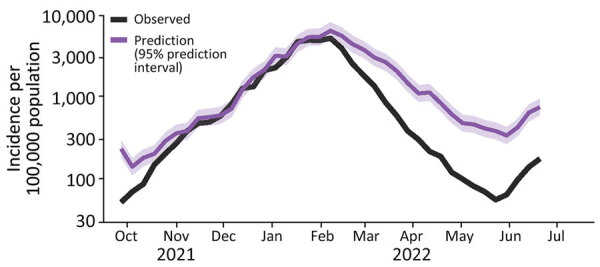
Predicted COVID-19 incidence at a constant testing rate (purple) based on the national model, compared with observed incidence (black), in study of wastewater surveillance data as a predictor of COVID-19 incidence, Denmark. The prediction is an estimate of the true incidence. The proportion of estimated true cases captured decreased from >80% to ≈20% during 2022.

We used the national model to estimate the COVID-19 incidence that would have been observed if the testing rate had remained constant (Figure 3). We used the highest testing rate in our study period (270 weekly tests/1,000 persons, as in the week of January 17, 2022). The difference between the model predictions and the observed incidence can be used as a measure of underreporting. Given the estimate from serologic studies that 70% of actual cases were captured in early 2022, we estimate that ≈15% of actual cases were captured by the national PCR testing system from April 2022 on.

## Discussion

We constructed a model to explain the observed incidence of COVID-19 in Denmark using wastewater data, information on the circulating variants, and the number of human tests performed as predictors. We found that we could accurately reconstruct the observed incidence curve. Results were consistent at a regional level and among the subgroup of frequently tested care personnel. Using data from a country with extensive wastewater and human testing systems, we demonstrated that predicting incidence based on wastewater surveillance is possible. We used these results to predict the incidence that could have been observed in Denmark if testing activity had remained high. In Denmark, after mass-testing programs were rolled back in the spring of 2022, wastewater analysis became a key source of information for the healthcare system in its handling of the COVID-19 pandemic.

The steeper association between wastewater and incidence during the Omicron period than in the Delta period might indicate that shedding dynamics differ between variants. However, the transition from Delta to Omicron coincided with the peak of rollout of vaccine booster doses (*22*). Information is lacking on how vaccination affects fecal shedding, but nasopharyngeal viral loads appear lower among vaccinated persons (*2*). Further studies are needed on how fecal shedding is affected by SARS-CoV-2 variants and vaccination status.

The model fit at the regional level was generally very similar to that for national results, although wastewater concentrations had less effect in the model for the Capital region. A likely factor is that the Capital region is dominated by 4 very large catchment areas, and correlations between wastewater concentration and incidence are poorer in larger catchment areas (*1*). Commuting across catchment areas, which is typical for the densely populated Capital region, might also have played a role.

We performed a subanalysis of healthcare personnel, who were consistently tested at a higher rate than the general population for screening purposes. We reasoned that the incidence of healthcare personnel would be less dependent on testing rates than the observed incidence in the general population. In fact, testing among this group followed a similar pattern over time to that of the general population and gave similar model results, possibly because the overall testing rate in Denmark was so high.

We used the national model to estimate what observed incidence would have been if the testing rate had remained at its maximum. We estimated that the proportion of actual cases identified had fallen to ≈15% by April 2022. However, if tests became more targeted over time as recommendations for regular screening tests were relaxed, the percentage of cases identified could be higher.

SARS-CoV-2 is known to decay faster at higher temperatures (*12*,*13*), but including an interaction term between wastewater results and temperature did not improve our model fit. The effect of temperature might have been outweighed by other unmeasured factors that affect SARS-CoV-2 decay, such as retention time in the sewage system or other chemical components of wastewater.

Unlike other studies (*1*), we did not find wastewater results to be a leading indicator of incidence. That difference might be because our analysis was based on weekly data, so we could only assess lag times in 7-day intervals. However, a lag of <1 week would likely have limited effects on public health action in practice. In addition, extensive human testing occurred during most of the study period, so infections might have been detected earlier than in other settings.

In our models, the coefficient for wastewater concentrations was <1. This result might seem surprising, because it means that a doubling in wastewater concentrations is not associated with a doubling in incidence (after adjusting for testing rate). Several explanations exist for this finding. First, the variability in the number of viral copies shed is best described by a log-normal distribution (*23*,*24*). The cumulative number of copies shed by a population will therefore follow a highly skewed distribution (*24*,*25*), which in itself is expected to lead to a coefficient <1, as seen in simulation models generating the relationship between the number of infected persons in the population and concentration of RNA in sewage (*26*). Second, observed incidence depends on the testing pattern; specifically, the probability that an infected person will be tested and have a positive result. The testing rate that we included in our models is an imperfect measure of this probability. Third, testing rate itself is influenced by incidence. Testing rates were highest, on average, when incidence was high. This factor might have increased the predictive power of testing rate in our models and therefore disadvantaged wastewater as a predictor. This interpretation is supported by the fact that the coefficient for testing rate in the national model (0.87 [95% CI 0.81–0.94]) is higher than the 0.7 that was found in Denmark’s method for estimating the reproduction number in the fall of 2020 (*27*). The secondary analysis of healthcare personnel provides further support. We expected observed incidence in this population to be a closer reflection of actual incidence (compared to national observed incidence numbers) because the testing rate was more stable. That expectation is consistent with our results: a larger coefficient for wastewater concentrations and marginally smaller (though with overlapping 95% CIs) coefficient for testing rate.

The first limitation of our model is that testing rate is influenced by incidence and changes in recommendations. However, this effect was likely smaller in our setting than in most others because there were high numbers of screening tests for asymptomatic people for much of the study period. Another limitation is that the performance of the model on the validation data was mixed. Validation model estimates clearly followed the same pattern as the observed incidence, but they were lower than the observed data; most of the 95% prediction intervals did not include the observed data. This discrepancy is likely because of the changes in recommendations in the first half of 2022, in which a gradual shift occurred toward less testing for screening purposes and a larger share of diagnostic testing of symptomatic persons. Finally, we could not incorporate the unknown effect of immunity (through vaccination or previous infection) on fecal shedding.

This study benefited from copious wastewater testing data because of the extensive surveillance system in Denmark. One remaining question is how well wastewater data perform in less developed surveillance systems. Denmark’s surveillance was scaled down after this study period to incorporate fewer WWTPs and fewer weekly samples. Repeating this analysis once enough data has been collected under the new system might help answer that question.

In conclusion, we performed a large-scale study of the association between wastewater results and observed incidence of COVID-19. Our relatively simple model makes it easy to specifically examine the association between wastewater results and incidence. We found that wastewater testing results can be used to accurately model the observed incidence of COVID-19, in combination with data on human tests. This finding implies that wastewater testing can serve as a proxy for incidence in the context of little to no human testing. The link between wastewater concentrations and incidence has been stronger since Omicron has been dominant. We found no effect of temperature on the association. For a wastewater surveillance system as extensive as that of Denmark, we believe wastewater results are a trustworthy indicator of actual incidence, especially in a situation in which human testing rates continue to decline.

Appendix Additional information about predicting COVID-19 incidence using wastewater surveillance data, Denmark, October 2021–June 2022
