# Supplementary material for: Predicting COVID-19 Incidence Using Wastewater Surveillance Data, Denmark, October 2021–June 2022
Source: Emerg Infect Dis. 2023 Aug;29(8):1589–97. doi: 10.3201/eid2908.221634 (PMC10370843; doi:10.3201/eid2908.221634)
Supplement: Appendix — Additional information about predicting COVID-19 incidence using wastewater surveillance data, Denmark, October 2021–June 2022 [file 22-1634-Techapp-s1.pdf]

Article DOI: https://doi.org/10.3201/eid2908.221634

EID cannot ensure accessibility for supplementary materials supplied by authors. Readers who have difficulty accessing supplementary content should contact the authors for assistance.

Predicting COVID-19 Incidence Using Wastewater Surveillance Data, Denmark, October 2021–June 2022

Appendix

R Session Info

```
─ Session info ────────────────────────────────────────────────────
setting  value
version  R version 4.2.3 (2023-03-15)
os       macOS Monterey 12.3.1
system   aarch64, darwin20
ui       X11
language (EN)
collate  en_US.UTF-8
ctype    en_US.UTF-8
tz       Europe/London
date     2023-04-19
pandoc   2.19.2 @
/Applications/RStudio.app/Contents/Resources/app/quarto/bin/tools/ (via rmarkdown)

─ Packages─────────────────────────────────────────
package              * version    date (UTC) lib source
abind                  1.4-5      2016-07-21 [1] CRAN (R 4.2.0)
anytime                0.3.9      2020-08-27 [1] CRAN (R 4.2.0)
assertthat           * 0.2.1      2019-03-21 [1] CRAN (R 4.2.0)
backports              1.4.1      2021-12-13 [1] CRAN (R 4.2.0)
base64enc              0.1-3      2015-07-28 [1] CRAN (R 4.2.0)
```

```
boot                 1.3-28.1   2022-11-22 [1] CRAN (R 4.2.3)
broom                1.0.4      2023-03-11 [1] CRAN (R 4.2.0)
broom.helpers        1.13.0     2023-03-28 [1] CRAN (R 4.2.0)
bslib                0.3.1      2021-10-06 [1] CRAN (R 4.2.0)
cachem               1.0.7      2023-02-24 [1] CRAN (R 4.2.0)
cellranger           1.1.0      2016-07-27 [1] CRAN (R 4.2.0)
checkmate            2.1.0      2022-04-21 [1] CRAN (R 4.2.0)
class                7.3-21     2023-01-23 [1] CRAN (R 4.2.3)
classInt             0.4-9      2023-02-28 [1] CRAN (R 4.2.0)
cli                * 3.6.1      2023-03-23 [1] CRAN (R 4.2.0)
clipr                0.8.0      2022-02-22 [1] CRAN (R 4.2.0)
clisymbols           1.2.0      2017-05-21 [1] CRAN (R 4.2.0)
cluster              2.1.4      2022-08-22 [1] CRAN (R 4.2.3)
colorspace           2.0-3      2022-02-21 [1] CRAN (R 4.2.0)
conflicted         * 1.2.0      2023-02-01 [1] CRAN (R 4.2.0)
crayon               1.5.1      2022-03-26 [1] CRAN (R 4.2.0)
curl                 4.3.2      2021-06-23 [1] CRAN (R 4.2.0)
data.table           1.14.2     2021-09-27 [1] CRAN (R 4.2.0)
DBI                  1.1.3      2022-06-18 [1] CRAN (R 4.2.0)
deldir               1.0-6      2021-10-23 [1] CRAN (R 4.2.0)
desc                 1.4.2      2022-09-08 [1] CRAN (R 4.2.0)
details            * 0.3.0      2022-03-27 [1] CRAN (R 4.2.0)
digest               0.6.29     2021-12-01 [1] CRAN (R 4.2.0)
distributional       0.3.2      2023-03-22 [1] CRAN (R 4.2.0)
dplyr              * 1.1.1      2023-03-22 [1] CRAN (R 4.2.0)
e1071                1.7-13     2023-02-01 [1] CRAN (R 4.2.0)
ellipsis             0.3.2      2021-04-29 [1] CRAN (R 4.2.0)
evaluate             0.15       2022-02-18 [1] CRAN (R 4.2.0)
fable              * 0.3.3      2023-03-22 [1] CRAN (R 4.2.0)
fabletools         * 0.3.3      2023-04-04 [1] CRAN (R 4.2.0)
fansi                1.0.3      2022-03-24 [1] CRAN (R 4.2.0)
farver               2.1.0      2021-02-28 [1] CRAN (R 4.2.0)
fastmap              1.1.0      2021-01-25 [1] CRAN (R 4.2.0)
```

```
feasts          0.3.1     2023-03-22 [1] CRAN (R 4.2.0)
forcats       * 0.5.1     2021-01-27 [1] CRAN (R 4.2.0)
foreign         0.8-84    2022-12-06 [1] CRAN (R 4.2.3)
Formula         1.2-5     2023-02-24 [1] CRAN (R 4.2.0)
gdata           2.18.0.1  2022-05-10 [1] CRAN (R 4.2.0)
generics        0.1.2     2022-01-31 [1] CRAN (R 4.2.0)
ggplot2       * 3.3.6     2022-05-03 [1] CRAN (R 4.2.0)
ggthemes      * 4.2.4     2021-01-20 [1] CRAN (R 4.2.0)
glue          * 1.6.2     2022-02-24 [1] CRAN (R 4.2.0)
goftest         1.2-3     2021-10-07 [1] CRAN (R 4.2.0)
gridExtra       2.3       2017-09-09 [1] CRAN (R 4.2.0)
gt              0.9.0     2023-03-31 [1] CRAN (R 4.2.0)
gtable          0.3.0     2019-03-25 [1] CRAN (R 4.2.0)
gtools          3.9.4     2022-11-27 [1] CRAN (R 4.2.0)
gtsummary       1.7.0     2023-01-13 [1] CRAN (R 4.2.0)
here          * 1.0.1     2020-12-13 [1] CRAN (R 4.2.0)
highr           0.9       2021-04-16 [1] CRAN (R 4.2.0)
Hmisc           5.0-1     2023-03-08 [1] CRAN (R 4.2.0)
hms             1.1.1     2021-09-26 [1] CRAN (R 4.2.0)
htmlTable       2.4.1     2022-07-07 [1] CRAN (R 4.2.0)
htmltools       0.5.5     2023-03-23 [1] CRAN (R 4.2.0)
htmlwidgets     1.6.2     2023-03-17 [1] CRAN (R 4.2.0)
httr            1.4.3     2022-05-04 [1] CRAN (R 4.2.0)
insight         0.19.1    2023-03-18 [1] CRAN (R 4.2.0)
ISOweek       * 0.6-2     2011-09-07 [1] CRAN (R 4.2.0)
janitor       * 2.2.0     2023-02-02 [1] CRAN (R 4.2.0)
jquerylib       0.1.4     2021-04-26 [1] CRAN (R 4.2.0)
jsonlite        1.8.0     2022-02-22 [1] CRAN (R 4.2.0)
KernSmooth      2.23-20   2021-05-03 [1] CRAN (R 4.2.3)
knitr           1.39      2022-04-26 [1] CRAN (R 4.2.0)
labeling        0.4.2     2020-10-20 [1] CRAN (R 4.2.0)
lattice         0.20-45   2021-09-22 [1] CRAN (R 4.2.3)
lifecycle       1.0.3     2022-10-07 [1] CRAN (R 4.2.0)
```

```
lme4                   1.1-32     2023-03-14 [1] CRAN (R 4.2.0)
lubridate            * 1.8.0      2021-10-07 [1] CRAN (R 4.2.0)
magrittr             * 2.0.3      2022-03-30 [1] CRAN (R 4.2.0)
MASS                   7.3-58.2   2023-01-23 [1] CRAN (R 4.2.3)
Matrix                 1.5-3      2022-11-11 [1] CRAN (R 4.2.3)
memoise                2.0.1      2021-11-26 [1] CRAN (R 4.2.0)
mgcv                   1.8-42     2023-03-02 [1] CRAN (R 4.2.3)
mice                   3.15.0     2022-11-19 [1] CRAN (R 4.2.0)
minqa                  1.2.5      2022-10-19 [1] CRAN (R 4.2.0)
munsell                0.5.0      2018-06-12 [1] CRAN (R 4.2.0)
nlme                 * 3.1-162    2023-01-31 [1] CRAN (R 4.2.3)
nloptr                 2.0.3      2022-05-26 [1] CRAN (R 4.2.0)
nnet                   7.3-18     2022-09-28 [1] CRAN (R 4.2.3)
numDeriv               2016.8-1.1 2019-06-06 [1] CRAN (R 4.2.0)
pacman                 0.5.1      2019-03-11 [1] CRAN (R 4.2.0)
patchwork            * 1.1.2      2022-08-19 [1] CRAN (R 4.2.0)
performance          * 0.10.3     2023-04-07 [1] CRAN (R 4.2.0)
PerformanceAnalytics * 2.0.4      2020-02-06 [1] CRAN (R 4.2.0)
pillar                 1.9.0      2023-03-22 [1] CRAN (R 4.2.0)
pkgconfig              2.0.3      2019-09-22 [1] CRAN (R 4.2.0)
png                    0.1-8      2022-11-29 [1] CRAN (R 4.2.0)
polyclip               1.10-4     2022-10-20 [1] CRAN (R 4.2.0)
progressr              0.13.0     2023-01-10 [1] CRAN (R 4.2.0)
proxy                  0.4-27     2022-06-09 [1] CRAN (R 4.2.0)
purrr                * 0.3.4      2020-04-17 [1] CRAN (R 4.2.0)
quadprog               1.5-8      2019-11-20 [1] CRAN (R 4.2.0)
Quandl                 2.11.0     2021-08-11 [1] CRAN (R 4.2.0)
quantmod             * 0.4.22     2023-04-07 [1] CRAN (R 4.2.0)
R6                     2.5.1      2021-08-19 [1] CRAN (R 4.2.0)
RColorBrewer           1.1-3      2022-04-03 [1] CRAN (R 4.2.0)
Rcpp                   1.0.10     2023-01-22 [1] CRAN (R 4.2.0)
readr                * 2.1.2      2022-01-30 [1] CRAN (R 4.2.0)
readxl               * 1.4.0      2022-03-28 [1] CRAN (R 4.2.0)
```

```
rlang               1.1.0    2023-03-14 [1] CRAN (R 4.2.0)
rmarkdown           2.14     2022-04-25 [1] CRAN (R 4.2.0)
rpart             * 4.1.19   2022-10-21 [1] CRAN (R 4.2.3)
rprojroot           2.0.3    2022-04-02 [1] CRAN (R 4.2.0)
rstudioapi          0.13     2020-11-12 [1] CRAN (R 4.2.0)
sass                0.4.5    2023-01-24 [1] CRAN (R 4.2.0)
scales            * 1.2.1    2022-08-20 [1] CRAN (R 4.2.0)
sessioninfo         1.2.2    2021-12-06 [1] CRAN (R 4.2.0)
sf                * 1.0-12   2023-03-19 [1] CRAN (R 4.2.0)
snakecase           0.11.0   2019-05-25 [1] CRAN (R 4.2.0)
spatstat          * 3.0-3    2023-01-28 [1] CRAN (R 4.2.0)
spatstat.data     * 3.0-1    2023-03-12 [1] CRAN (R 4.2.0)
spatstat.explore  * 3.1-0    2023-03-14 [1] CRAN (R 4.2.0)
spatstat.geom     * 3.1-0    2023-03-12 [1] CRAN (R 4.2.0)
spatstat.linnet   * 3.1-0    2023-04-14 [1] CRAN (R 4.2.0)
spatstat.model    * 3.2-1    2023-02-15 [1] CRAN (R 4.2.0)
spatstat.random   * 3.1-4    2023-03-13 [1] CRAN (R 4.2.0)
spatstat.sparse     3.0-1    2023-03-12 [1] CRAN (R 4.2.0)
spatstat.utils      3.0-2    2023-03-11 [1] CRAN (R 4.2.0)
stringi             1.7.6    2021-11-29 [1] CRAN (R 4.2.0)
stringr           * 1.4.0    2019-02-10 [1] CRAN (R 4.2.0)
tensor              1.5      2012-05-05 [1] CRAN (R 4.2.0)
tibble              3.2.1    2023-03-20 [1] CRAN (R 4.2.0)
tidylog             1.0.2    2020-07-03 [1] CRAN (R 4.2.0)
tidyquant         * 1.0.7    2023-03-31 [1] CRAN (R 4.2.0)
tidyr             * 1.2.0    2022-02-01 [1] CRAN (R 4.2.0)
tidyselect          1.2.0    2022-10-10 [1] CRAN (R 4.2.0)
tsibble             1.1.3    2022-10-09 [1] CRAN (R 4.2.0)
TTR               * 0.24.3   2021-12-12 [1] CRAN (R 4.2.0)
tzdb                0.3.0    2022-03-28 [1] CRAN (R 4.2.0)
units               0.8-1    2022-12-10 [1] CRAN (R 4.2.0)
urca                1.3-3    2022-08-29 [1] CRAN (R 4.2.0)
utf8                1.2.2    2021-07-24 [1] CRAN (R 4.2.0)
```

```
vctrs                 0.6.2      2023-04-19 [1] CRAN (R 4.2.3)
weights               1.0.4      2021-06-10 [1] CRAN (R 4.2.0)
withr                 2.5.0      2022-03-03 [1] CRAN (R 4.2.0)
writexl             * 1.4.2      2023-01-06 [1] CRAN (R 4.2.0)
xfun                  0.38       2023-03-24 [1] CRAN (R 4.2.0)
xml2                  1.3.3      2021-11-30 [1] CRAN (R 4.2.0)
xts                 * 0.13.1     2023-04-16 [1] CRAN (R 4.2.0)
yaml                  2.3.5      2022-02-21 [1] CRAN (R 4.2.0)
zoo                 * 1.8-12     2023-04-13 [1] CRAN (R 4.2.0)
[1] /Library/Frameworks/R.framework/Versions/4.2-arm64/Resources/library
```

Model Selection

We tested the following model specifications to model national incidence. Model 5 is the model that we ultimately selected.

1. wastewater concentration + AR(1)

2. wastewater concentration + testing rate + AR(1)

3. wastewater concentration + testing rate + wastewater concentration * wastewater temperature + AR(1)

4. wastewater concentration + testing rate + wastewater concentration * wastewater temperature + wastewater concentration * Delta (%) + AR(1)

5. wastewater concentration + testing rate + wastewater concentration * Delta (%) + AR(1)

6. wastewater concentration + wastewater concentration * Delta (%) + AR(1)

7. testing rate + AR(1)

8. wastewater concentration + testing rate + wastewater concentration * Delta (%) + testing rate * Delta (%) + AR(1)

The log-likelihood, AIC, and BIC measures for each tested model are in Appendix Table 2.

Model Equations

In the national model, incidence (*I*) is modeled as:

$$I = 10^{0.43*\log_{10}(\boldsymbol{WW})+0.86*\log_{10}(\boldsymbol{T})-\ 0.16*\log_{10}(\boldsymbol{WW})*\boldsymbol{P_D}+\ 0.43*\ AR(1)-0.14}$$

WW = weekly wastewater result (copies per person)

T = weekly testing rate (tests / 1,000 population)

P_D = proportion of human samples that were of the Delta variant (%)

AR(1) = autoregressive coefficient of order 1

The care personnel and regional models follow the same structure.

Appendix Table 1. Wastewater treatment plant characteristics

Treatment plant	Population	Samples included	RNA copies per liter[1]		24-h flow (liters)[1]		Temperature[2]	
			Median	IQR	Median	IQR	Median	IQR
Region Hovedstaden								
Dragør	14,582	74	4.26	1.28	3.73	0.20	4.00	1.00
Fredensborg/Lønholt	10,272	98	4.32	0.88	3.41	0.13	10.40	4.70
Frederikssund	20,767	89	4.07	0.83	3.77	0.14	1.00	0.00
Gilleleje	8,383	73	3.97	0.71	3.56	0.24	11.60	5.35
Halsnæs (Melby)	30,110	98	4.17	0.92	3.95	0.17	10.70	4.90
Helsinge	13,585	74	4.10	0.84	3.79	0.16	11.80	5.80
Helsingør	30,752	92	4.46	0.60	3.80	0.12	13.20	3.43
Hillerød	39,918	104	4.00	0.73	4.27	0.24	10.25	4.88
Humlebæk/Nivå	18,330	94	4.46	0.75	3.62	0.18	11.90	4.30
Hvidovre (Avedøre)	252,811	93	4.00	1.05	4.79	0.14	4.00	2.00
København (Damhusåen)	290,219	97	4.40	0.65	4.82	0.22	5.00	2.20
Lillerød	17,447	94	4.31	0.85	3.66	0.11	10.70	4.97
Lynetten (nordre tilløb)	211,322	99	4.40	0.61	4.77	0.19	4.00	7.20
Lynetten (søndre tilløb)	430,548	92	4.47	0.59	4.94	0.07	4.00	4.10
Lyngby-Taarbæk	55,224	92	4.23	0.64	4.45	0.17	12.00	7.00
Lynge	4,709	98	4.01	0.94	2.99	0.25	9.45	5.95
Måløv	50,072	99	4.35	0.76	3.96	0.19	11.50	5.10
Neder Dråby	3,554	87	2.12	1.15	2.92	0.22	1.00	0.00
Nexø	4,714	105	3.39	1.17	3.58	0.40	9.85	5.35
Nordkysten	12,122	102	4.04	0.82	3.72	0.28	10.00	4.00
Ølstykke	16,622	96	4.12	1.01	3.53	0.16	1.00	0.00
Rønne	18,640	108	3.78	0.99	3.91	0.29	11.45	4.02
Sjælsø	13,262	104	4.09	1.00	3.50	0.30	11.00	4.63
Slangerup	7,465	87	3.20	1.62	3.20	0.18	1.00	0.00
Stavnsholt	20,554	91	4.41	0.87	3.51	0.10	12.70	4.75
Stenløse	12,167	92	4.46	0.83	3.28	0.16	1.00	0.00
Sydkysten	18,485	100	3.95	0.89	3.78	0.18	10.45	4.70
Tårnby	42,415	83	4.22	0.69	4.08	0.25	12.70	4.25
Tejn	2,549	105	3.48	1.26	3.37	0.42	7.85	5.50
Tørslev	3,852	91	4.08	1.19	3.13	0.20	1.00	0.00
Usserød	35,488	93	3.92	1.09	3.97	0.15	11.30	4.40
Vedbæk	11,926	103	4.13	0.96	3.69	0.21	10.50	4.45
Region Midtjylland								
Aarhus (Åby)	59,770	106	4.20	1.10	4.10	0.24	12.90	4.70
Aarhus (Egå)	86,360	103	4.14	1.15	4.25	0.16	12.00	3.50
Aarhus (Marselisborg)	122,064	102	4.19	1.24	4.40	0.14	14.20	4.00
Aarhus (Viby)	79,099	108	4.09	1.05	4.25	0.28	10.85	4.23
Aulum	5,751	105	3.93	1.59	3.26	0.25	9.50	4.00
Bjerringbro	11,328	93	3.85	1.29	3.74	0.16	10.30	4.50

Treatment plant	Population	Samples included	RNA copies per liter[1]		24-h flow (liters)[1]		Temperature[2]	
			Median	IQR	Median	IQR	Median	IQR
Boeslum	7,984	93	4.14	0.87	3.33	0.08	5.00	2.00
Brædstrup	10,415	102	4.02	1.11	3.44	0.31	8.85	4.90
Brande	8,190	109	3.39	1.30	3.65	0.18	12.00	3.30
Drøsbro	2,103	25	3.80	1.14	2.86	0.12	10.10	3.30
Fornæs	21,348	107	3.15	1.36	4.09	0.18	11.17	3.62
Hadsten	10,389	33	3.91	1.47	3.50	0.28	9.40	5.50
Hammel	8,994	31	4.49	1.35	3.21	0.19	9.45	5.15
Harboøre	5,194	99	3.98	1.31	3.38	0.16	16.00	5.00
Harre-Vejle	3,134	101	3.20	1.34	3.31	0.25	8.60	4.30
Hedensted	20,286	100	3.77	1.00	3.90	0.15	11.00	4.00
Herning	55,728	103	3.92	1.00	4.39	0.29	11.00	3.50
Hinnerup	13,717	33	4.48	1.17	3.48	0.18	9.90	3.80
Holstebro øst	11,480	100	4.03	1.05	3.84	0.14	11.30	3.62
Holstebro vest	30,431	92	3.47	1.27	3.74	0.09	15.20	3.40
Hørning	8,917	78	4.25	0.83	3.26	0.28	9.90	4.30
Horsens	78,844	96	4.14	0.87	4.39	0.14	14.05	3.93
Hvide Sande	2,987	85	3.62	1.67	3.18	0.38	9.30	4.87
Ikast	22,195	107	4.03	1.03	3.88	0.26	8.00	5.80
Juelsminde	8,161	95	4.18	1.03	3.38	0.16	10.00	4.50
Karup	5,121	37	3.03	2.01	3.31	0.38	11.10	5.80
Kjellerup	9,235	98	4.06	1.04	3.47	0.25	9.35	5.27
Langå	7,522	64	3.19	1.54	2.98	0.15	11.10	4.75
Lemvig	11,592	97	3.76	0.88	3.60	0.22	10.30	3.60
Mørke	8,936	107	4.00	0.93	3.37	0.27	10.15	4.92
Nørre Snede	4,991	102	4.00	0.63	3.02	0.18	10.15	3.52
Odder	20,050	102	4.14	0.96	3.69	0.18	10.65	4.95
Randers Nord	29,990	100	4.11	1.02	3.78	0.15	12.00	3.95
Randers Syd	38,651	106	4.15	1.11	3.90	0.15	9.20	4.00
Randers Vest	21,472	94	3.93	0.97	3.64	0.23	9.95	4.28
Ringkøbing	13,518	22	3.95	1.86	3.81	0.15	7.20	5.70
Ry	7,876	83	4.42	0.93	3.11	0.24	10.50	5.00
Skanderborg	24,918	79	4.12	0.92	3.75	0.19	11.70	4.10
Skive	33,048	107	3.75	1.04	4.19	0.27	9.00	3.75
Skjern/Tarm	17,248	104	3.81	1.52	3.91	0.18	13.12	4.28
Skovby	14,646	81	3.89	0.85	3.66	0.38	9.40	4.60
Søholt	59,148	91	4.30	0.94	4.18	0.10	11.50	3.90
Struer	14,961	103	4.08	0.88	3.66	0.20	10.90	4.70
Sunds	5,195	104	3.66	1.39	3.61	0.18	10.00	2.70
Them	5,296	95	3.83	1.03	3.24	0.11	11.70	4.60
Trehøje Øst	5,545	106	3.63	1.59	3.45	0.26	9.50	3.50
Truust	4,823	93	3.94	0.97	3.15	0.15	9.00	4.30
Viborg	48,974	88	4.19	0.98	4.03	0.21	10.55	4.50
Videbæk	7,078	105	3.94	1.11	3.37	0.27	9.50	3.50
Vinderup	5,097	101	3.93	1.29	3.45	0.24	9.80	4.00
Region Nordjylland								
Aalborg Øst	68,413	104	4.00	0.75	4.24	0.17	12.00	3.05
Aalborg Vest	141,626	102	3.97	0.83	4.65	0.16	11.25	3.27
Aars	9,972	107	4.01	1.05	3.57	0.08	5.00	0.00
Attrup	5,625	101	2.93	1.65	3.52	0.25	8.86	5.30
Brønderslev	16,246	85	3.92	0.74	3.72	0.22	10.00	2.00
Fjerritslev	5,622	100	3.93	1.11	3.33	0.19	9.44	4.50
Frederikshavn	30,415	107	3.97	1.06	4.12	0.20	11.60	4.88
Hanstholm	3,624	93	4.45	1.03	2.75	0.12	7.00	4.62
Hirtshals	10,394	109	3.82	1.38	3.82	0.21	9.70	4.30
Hjørring	33,814	102	4.11	1.03	4.04	0.28	9.85	4.15
Karby	1,039	91	3.17	1.87	3.11	0.19	8.40	5.45
Løgstør	6,580	104	3.63	1.18	3.50	0.15	5.00	0.00
Løkken (Nr. Lyngby)	3,404	103	3.61	1.23	3.30	0.21	9.50	4.40
Mariagerfjord	34,566	104	3.85	1.13	4.09	0.15	10.90	4.31
Nykøbing Mors	15,136	104	3.83	1.04	3.70	0.21	10.75	5.00
Sæby	11,987	94	3.71	1.24	3.83	0.11	14.80	4.75
Sigsgård	18,307	106	4.03	1.11	3.77	0.26	11.50	5.35

Treatment plant	Population	Samples included	RNA copies per liter[1]		24-h flow (liters)[1]		Temperature[2]	
			Median	IQR	Median	IQR	Median	IQR
Sindal	4,839	108	3.63	1.37	3.25	0.19	9.15	3.97
Skagen	7,497	104	3.35	0.92	4.01	0.17	13.50	3.30
Stistrup	11,422	105	4.32	1.07	3.31	0.11	5.00	0.00
Sundby (Morsø)	687	90	2.98	1.84	2.43	0.40	9.00	3.00
Tåbel	6,746	107	3.51	0.91	3.49	0.40	9.40	4.07
Thisted	16,182	106	3.48	1.17	3.96	0.25	17.20	3.98
Vilsund	4,411	102	3.54	1.44	3.32	0.43	7.00	4.00
Region Sjælland								
Bjergmarken	64,493	92	2.78	1.27	4.19	0.18	13.50	4.00
Borup	5,662	106	3.86	0.81	3.35	0.33	9.40	5.68
Dalby	2,487	98	3.90	1.15	2.90	0.21	10.00	5.10
Fårevejle	10,969	98	3.92	0.97	3.48	0.14	11.50	4.97
Faxe	7,610	102	3.72	1.04	3.56	0.34	6.40	4.85
Fuglebjerg	5,816	102	4.08	1.15	3.09	0.33	9.60	4.90
Haslev	14,568	97	3.93	0.78	3.56	0.31	10.40	4.90
Holbæk	36,320	92	4.03	0.96	3.87	0.12	13.95	4.20
Holme Olstrup	7,656	106	4.00	0.98	3.34	0.39	9.95	5.20
Hvalsø	7,413	88	4.20	0.74	3.28	0.31	10.45	5.47
Jyllinge	13,332	100	4.38	0.88	3.32	0.11	12.75	5.60
Kalundborg	18,701	94	3.72	1.26	3.87	0.18	11.00	5.20
Køge	53,293	94	4.11	0.90	4.13	0.20	12.40	5.05
Korsør	20,474	74	4.24	0.67	3.66	0.14	11.65	4.77
Maribo (Hunseby)	17,296	108	3.96	1.20	3.81	0.35	10.30	4.30
Marielyst	4,321	65	4.43	0.80	3.22	0.15	7.60	1.30
Mosede	47,491	99	4.34	0.74	4.12	0.19	11.70	4.60
Næstved	69,102	107	4.02	0.85	4.32	0.28	10.60	4.31
Nakskov	12,862	109	3.80	0.91	3.83	0.22	11.70	5.60
Nykøbing	5,890	106	3.73	0.99	3.25	0.30	12.35	3.70
Nykøbing Falster	26,505	70	4.26	0.95	3.94	0.29	7.60	1.30
Ornum	8,808	108	3.71	1.11	3.56	0.32	9.90	5.45
Præstø	4,623	103	3.84	1.16	3.37	0.31	9.90	6.20
Ringsted C	31,851	102	3.70	1.08	3.97	0.27	13.70	6.03
Rødbyhavn	3,537	94	3.34	1.51	3.24	0.22	10.80	4.70
Skælskør	8,005	98	4.12	1.03	3.36	0.16	13.34	4.93
Slagelse	35,911	92	4.17	1.07	4.01	0.15	13.46	4.28
Solrød	19,197	100	4.08	0.81	3.70	0.17	11.30	4.00
Sorø	13,798	1	2.15	0.00	3.28	0.00	18.00	0.00
Stege	5,104	102	3.53	1.42	3.35	0.25	11.05	6.10
Store Heddinge	4,935	98	3.95	1.39	2.97	0.18	10.25	6.65
Strøby	9,888	106	4.10	0.96	3.47	0.32	10.05	6.02
Tornved	8,195	92	3.98	0.63	3.41	0.23	10.40	5.30
Tysinge	9,272	88	4.18	0.97	3.55	0.38	9.10	4.80
Viby	5,798	102	4.10	0.96	3.28	0.31	10.45	5.77
Vordingborg	22,542	101	4.00	0.96	3.79	0.20	11.70	5.20
Region Syddanmark								
Å Strand	2,628	43	3.77	1.05	3.07	0.21	7.75	2.85
Ærøskøbing	1,921	66	4.30	1.49	2.84	0.19	9.00	2.00
Årup	4,561	46	3.73	1.57	3.29	0.27	7.40	2.38
Assens	8,204	43	3.48	1.37	3.49	0.16	10.75	3.15
Bogense	7,170	99	4.10	1.14	3.33	0.15	11.10	4.00
Bov	6,233	98	3.79	0.88	3.33	0.30	9.10	4.15
Bramming Nord	5,035	104	3.95	1.26	3.05	0.37	10.80	3.30
Brejning	12,850	101	3.91	1.21	3.54	0.23	10.40	4.12
Brørup	5,337	101	3.87	1.44	3.42	0.42	9.00	7.62
Christiansfeld	5,580	101	3.60	1.27	3.40	0.18	12.28	4.06
Egebjerg Syd	4,113	103	3.45	1.87	3.20	0.27	9.80	3.75
Egsmade	40,860	98	3.79	1.09	4.10	0.22	11.20	3.85
Esbjerg Øst	24,278	107	3.94	1.44	4.11	0.19	11.40	4.40
Esbjerg Vest	61,935	102	3.67	0.96	4.39	0.11	13.95	2.50
Fåborg	21,569	3	4.23	1.01	4.37	0.05	8.40	0.80
Fredericia	51,429	94	3.87	1.14	4.28	0.16	14.35	3.66
Gaardeby	6,786	105	3.69	1.05	3.47	0.24	9.90	4.50

Treatment plant	Population	Samples included	RNA copies per liter[1]		24-h flow (liters)[1]		Temperature[2]	
			Median	IQR	Median	IQR	Median	IQR
Give	12,140	98	3.68	0.89	3.71	0.29	10.30	4.38
Gram	4,004	97	3.81	1.15	3.45	0.14	9.30	4.40
Grindsted/Billund	16,699	105	3.79	0.72	4.05	0.12	10.90	4.20
Haderslev	30,473	94	3.81	1.01	3.95	0.20	13.00	3.23
Haraldskær	15,179	100	3.86	0.80	3.58	0.19	11.80	4.67
Hårby	3,518	44	3.31	1.30	3.15	0.26	6.90	2.02
Hofmansgave	718	70	2.16	1.11	3.05	0.22	9.20	4.20
Holsted by	7,902	100	3.51	0.96	3.70	0.28	8.80	6.60
Kerteminde/Munkebo	15,807	105	3.89	0.87	3.85	0.23	10.60	4.70
Kolding	72,474	96	4.01	0.83	4.33	0.20	12.30	4.15
Kollund	2,418	94	4.06	1.17	2.75	0.16	9.40	3.90
Kværndrup	1,966	3	3.77	1.15	3.14	0.14	12.60	1.00
Middelfart	22,818	106	4.02	0.94	3.89	0.25	11.60	4.10
Nr. Åby	5,502	104	2.56	1.77	3.45	0.25	10.70	4.05
Nr. Nebel	2,412	99	3.96	1.37	3.21	0.25	8.90	4.45
Nyborg	30,414	5	3.62	0.38	3.94	0.16	14.00	0.00
Odense (Ejby Mølle)	133,463	103	3.79	1.50	4.68	0.17	11.60	3.45
Odense (Nordøst)	17,351	98	4.07	1.16	3.74	0.13	11.70	3.45
Odense (Nordvest)	50,344	107	4.03	1.02	4.21	0.20	11.50	4.10
Ørbæk	3,660	100	3.72	1.08	3.13	0.20	11.95	4.90
Otterup	10,401	101	3.90	0.81	3.55	0.18	10.20	4.50
Outrup	1,403	94	3.28	1.47	2.87	0.21	8.25	5.10
Ribe	12,900	107	3.78	1.08	3.74	0.29	9.80	4.15
Ringe	7,600	90	3.50	1.17	3.41	0.16	12.20	4.10
Rødding	8,664	105	3.57	1.14	3.40	0.38	7.80	6.05
Rudkøbing	5,403	97	3.86	1.12	3.19	0.26	11.90	4.50
Sdr. Nærå	8,059	7	4.09	0.45	3.37	0.21	10.10	3.15
Skærbæk	3,972	85	3.84	1.31	3.37	0.22	10.50	5.10
Skovlund	8,151	106	3.54	1.32	3.54	0.24	10.05	3.50
Sønderborg (Broager)	5,518	99	3.64	1.35	3.28	0.22	14.00	1.50
Sønderborg (Gråsten)	6,540	97	3.79	1.23	3.41	0.15	14.00	0.00
Sønderborg (Himmark)	10,524	103	3.96	1.02	3.52	0.28	11.00	3.30
Sønderborg	44,201	102	3.74	0.92	4.14	0.18	15.00	3.50
Søndersø	9,756	101	3.67	1.18	3.54	0.24	9.15	3.93
Stegholt	28,818	102	3.81	0.92	4.02	0.22	11.30	4.10
Strandgården	2,509	100	3.81	1.07	3.02	0.28	9.20	5.27
Tønder	10,514	109	3.80	1.09	3.81	0.18	14.20	4.80
Vamdrup	11,565	107	3.74	1.26	3.57	0.18	10.25	3.97
Varde	20,785	107	3.90	1.06	3.97	0.24	10.90	3.85
Vejen	20,652	101	3.63	0.76	3.91	0.27	8.25	6.48
Vejle	70,939	99	3.92	0.71	4.42	0.19	11.40	4.05
Vojens	7,910	90	3.99	1.05	3.28	0.18	10.60	4.70

[1] Calculated on the log_{10} scale.
[2] Contains missing values.

Appendix Table 2. Log-likelihood, AIC, and BIC measures for each tested model

Model	Log-likelihood	AIC	BIC
1	37.6	−67.3	−60.6
2	49.3	−88.6	−80.2
3	50.3	−88.6	−78.7
4	64.0	−113.9	−102.3
**5**	**63.1**	**−114.2**	**−104.3**
6	38.3	−66.6	−58.3
7	40.5	−73.0	−66.3
8	63.2	−112.3	−100.7

Appendix Table 3. Validation accuracy measures*

Model	Region	ME	RMSE	MAE	MPE	MAPE	ACF1	PI coverage
National	–	17.3	21.2	17.3	16.7	16.7	0.5	57%
Care personnel	–	−3.8	21.4	19.8	−7.5	17.7	0.1	100%
Regional	Capital Region	15.4	27.5	20.2	8.6	15.0	0.3	86%
Regional	Central Denmark	18.9	20.4	18.9	22.4	22.4	0.6	43%
Regional	North Denmark	16.6	21.1	16.6	18.9	18.9	0.4	57%
Regional	Southern Denmark	17.3	22.1	17.3	20.9	20.9	0.3	57%
Regional	Zealand	−1.0	14.9	10.6	0.3	9.8	−0.2	86%

*ACF1, autocorrelation at lag 1; MAE, mean absolute error; MAPE, mean absolute percent error; ME, mean error; MPE, mean percent error; PI coverage, percentage of validation observations contained in 95% prediction interval; RMSE, root mean squared error.

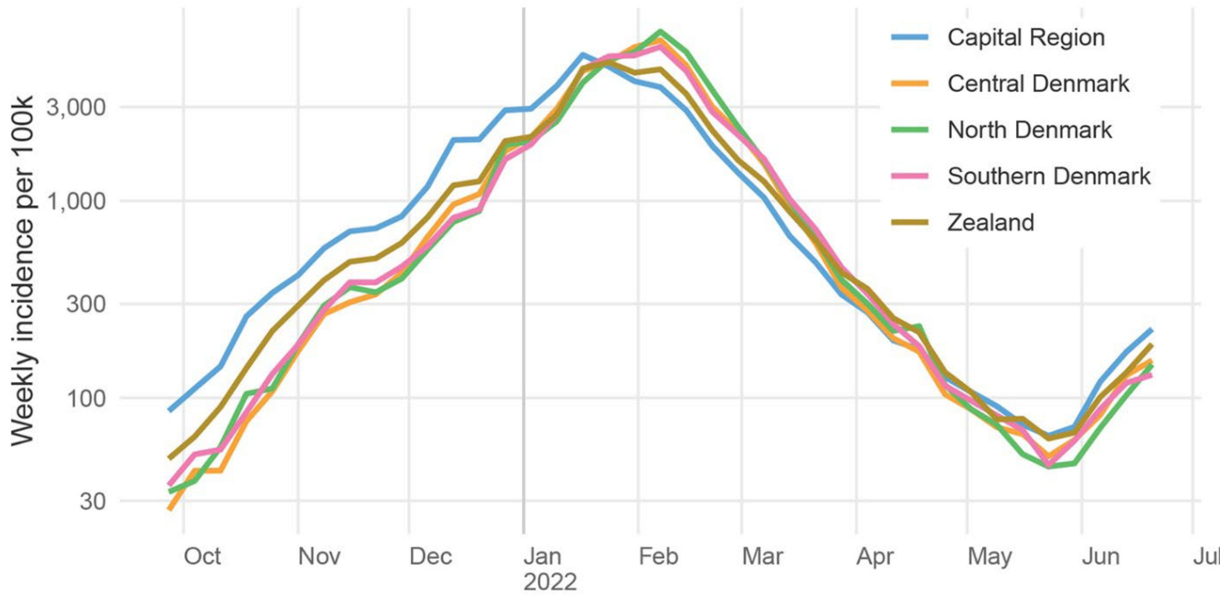

Appendix Figure 1. COVID-19 incidence over time by region

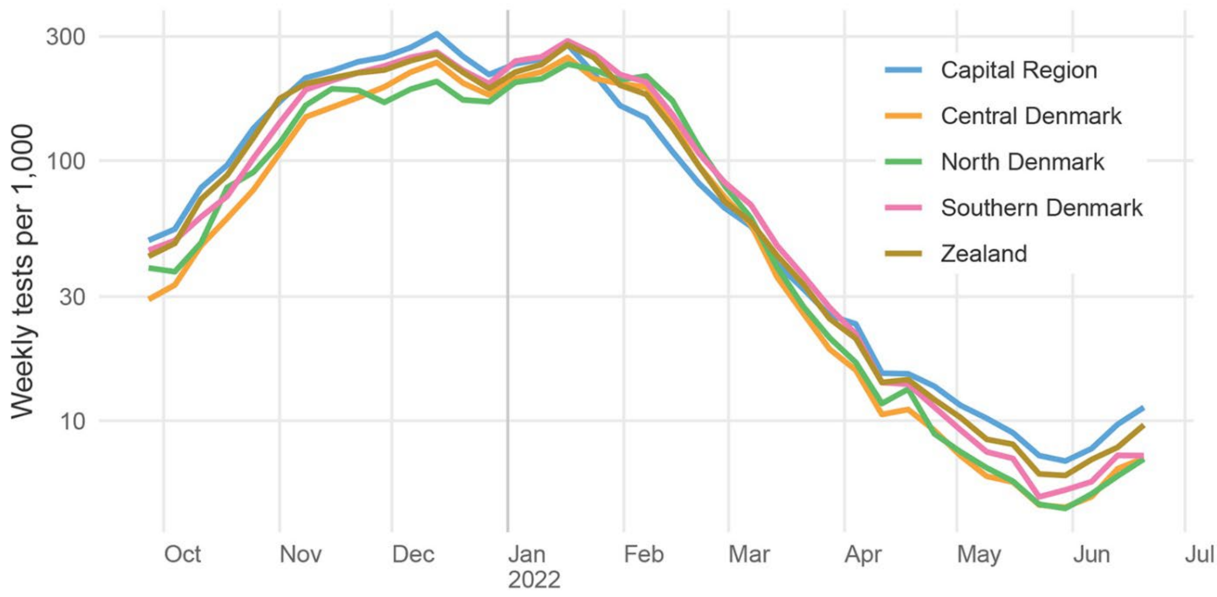

Appendix Figure 2. COVID-19 testing rate over time by region

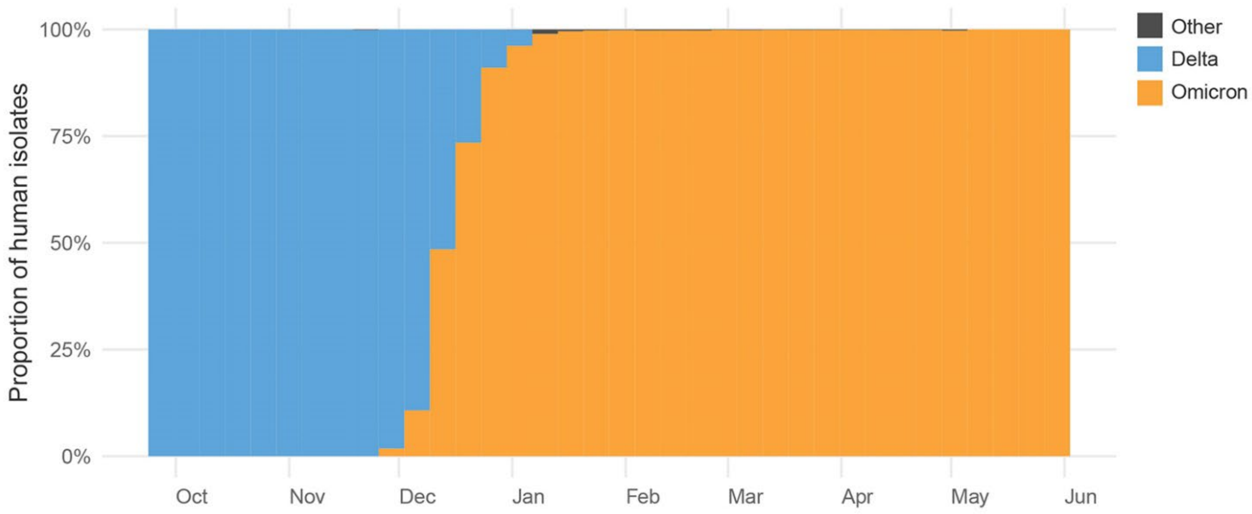

Appendix Figure 3. Variant proportion over time, based on human samples

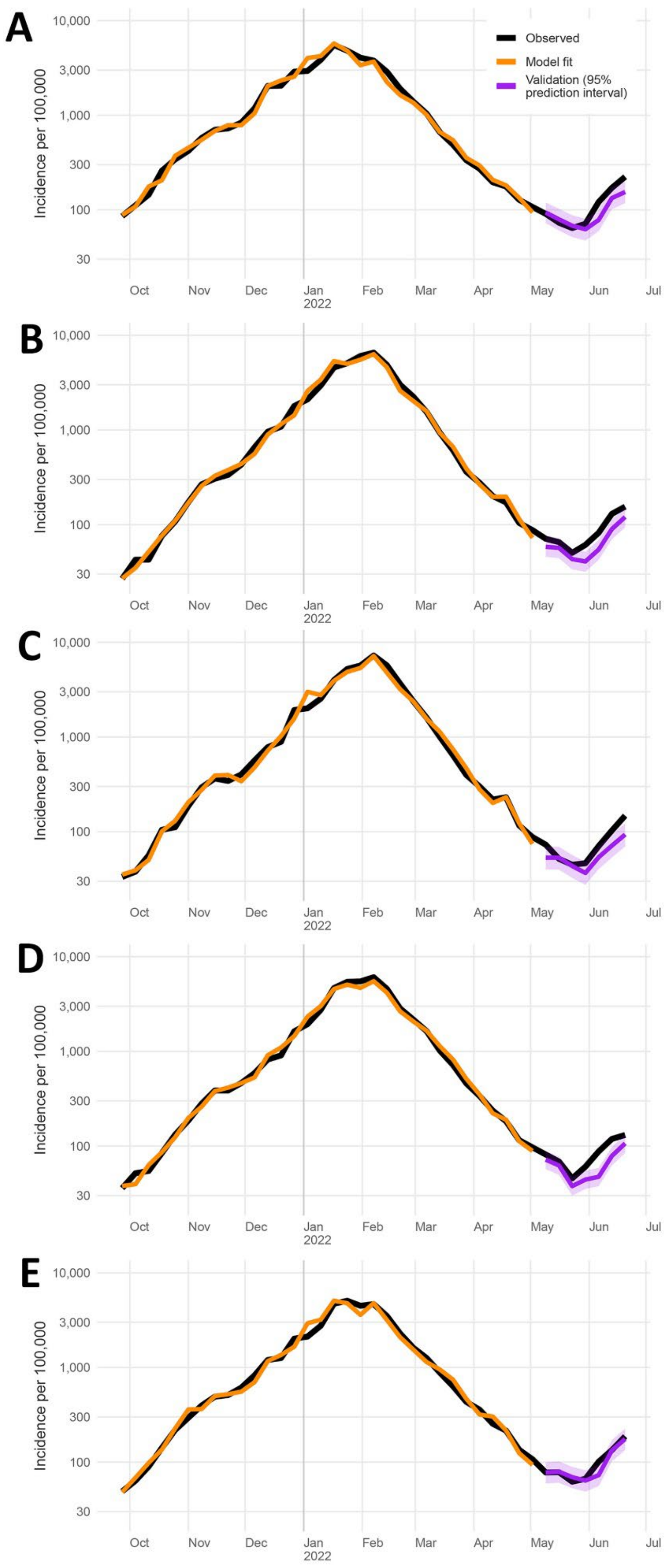

Appendix Figure 4. Model fit and forecasts based on the regional models. A) Capital Region, B) Central Denmark, C) North Denmark, D) Southern Denmark, E) Zealand.
